# RETRACTED: Cataldi et al. Neutral Sphingomyelinase Modulation in the Protective/Preventive Role of rMnSOD from Radiation-Induced Damage in the Brain. *Int. J. Mol. Sci.* 2019, *20*, 5431

**DOI:** 10.3390/ijms27062812

**Published:** 2026-03-20

**Authors:** Samuela Cataldi, Antonella Borrelli, Maria Rachele Ceccarini, Irina Nakashidze, Michela Codini, Oleg Belov, Alexander Ivanov, Eugene Krasavin, Ivana Ferri, Carmela Conte, Federica Filomena Patria, Giovanna Traina, Tommaso Beccari, Aldo Mancini, Francesco Curcio, Francesco Saverio Ambesi-Impiombato, Elisabetta Albi

**Affiliations:** 1 Department of Pharmaceutical Sciences, University of Perugia, 06126 Perugia, Italy; samuelacataldi@libero.it (S.C.); chele@hotmail.it (M.R.C.); irinanakashidze@yahoo.com (I.N.); michela.codini@unipg.it (M.C.); carmela.conte@unipg.it (C.C.); patriafederica@gmail.com (F.F.P.); giovanna.traina@unipg.it (G.T.); tommaso.beccari@unipg.it (T.B.); 2 Molecular Biology and Viral Oncology Unit, Istituto Nazionale Tumori IRCCS “Fondazione G. Pascale”, 80131 Napoli, Italy; a.borrelli@istitutotumori.na.it; 3 Laboratory of Radiation Biology, Joint Institute for Nuclear Research, 141980 Dubna, Russia; dem@jinr.ru (O.B.); a1931192@mail.ru (A.I.); krasavin@jinr.ru (E.K.); 4 Division of Pathological Anatomy and Histology, Department of Experimental Medicine, School of Medicine and Surgery, University of Perugia, 06126 Perugia, Italy; ivanaferri@gmail.com; 5 Laedhexa Biotechnologies Inc., San Francisco, CA QB3@953, USA; aldo_mancini@tiscali.it; 6 Dipartimento di Area Medica, University of Udine, 33100 Udine, Italy; francesco.curcio@uniud.it (F.C.); ambesis@me.com (F.S.A.-I.)

The journal retracts the article titled “Neutral Sphingomyelinase Modulation in the Protective/Preventive Role of rMnSOD from Radiation-Induced Damage in the Brain” [1], cited above.

Following publication, concerns were brought to the attention of the Editorial Office regarding the integrity of images presented in this publication [1].

In accordance with standard journal procedures, the Editorial Office and Editorial Board conducted an investigation that confirmed indications of inappropriate editing and duplications in Figures 2 and 3. Although the authors cooperated with the investigation, the explanations and supporting materials provided were not deemed sufficient by the Editorial Board to resolve the identified concerns via the requested correction. As a result, the Editorial Board has lost confidence in the reliability of the findings and decided to retract this publication [1], as per MDPI’s retraction policy.

This retraction was approved by the Editor-in-Chief of the journal *IJMS*.

The authors have been informed of this retraction and have indicated their disagreement with this decision.
